# Long-term consequences of reduced availability and compensatory supplementation of sialylated HMOs on cognitive capabilities

**DOI:** 10.3389/fncel.2023.1091890

**Published:** 2023-01-30

**Authors:** Edoardo Pisa, Alice Traversa, Viviana Caputo, Angela Maria Ottomana, Jonas Hauser, Simone Macrì

**Affiliations:** ^1^Centre for Behavioural Sciences and Mental Health, Istituto Superiore di Sanità, Rome, Italy; ^2^Laboratory of Clinical Genomics, Fondazione Istituto di Ricovero e Cura a Carattere Scientifico Casa Sollievo della Sofferenza, San Giovanni Rotondo, Italy; ^3^Department of Experimental Medicine, Sapienza University of Rome, Rome, Italy; ^4^Department of Medicine and Surgery, University of Parma, Parma, Italy; ^5^Brain Health, Nestlé Institute of Health Sciences, Nestlé Research, Société des Produits Nestlé Société Anonyme, Lausanne, Switzerland

**Keywords:** breast milk bioactive components, executive functions, memory, sialic acid, sialyllactose, breastfeeding, brain

## Abstract

Breast milk (BM) is the optimal source of nutrition for mammals’ early life. It exerts multiple benefits, including the development of cognitive capabilities and protection against several diseases like obesity and infection of the respiratory tract. However, which components of BM are involved in individual development has remained elusive. Sialylated human milk oligosaccharides (HMOs) may constitute a valid candidate, whereby they represent the principal source of sialic acid and act as building blocks for brain development. We hypothesize that the reduced availability of two HMOs, sialyl(alpha2,6)lactose (6′SL) and sialyl(alpha2,3)lactose (3′SL), may impair attention, cognitive flexibility, and memory in a preclinical model and that the exogenous supplementation of these compounds may contrast the observed deficits. We evaluated cognitive capabilities in a preclinical model exposed to maternal milk containing reduced concentrations of 6′SL and 3′SL during lactation. To modulate their concentrations, we utilized a preclinical model characterized by the absence of genes that synthesize 3′SL and 6′SL (B6.129-*St3gal4*^tm1.1Jxm^ and *St6gal1^tm2Jxm^*, double genetic deletion), producing milk lacking 3′SL and 6′SL. Then, to ensure exposure to 3′SL–6′SL-poor milk in early life, we adopted a cross-fostering protocol. The outcomes assessed in adulthood were different types of memory, attention and information processing, some of which are part of executive functions. Then, in the second study, we evaluated the long-term compensatory potential of the exogenous oral supplementation of 3′SL and 6′SL during lactation. In the first study, exposure to HMO-poor milk resulted in reduced memory and attention. Specifically, it resulted in impaired working memory in the T-maze test, in reduced spatial memory in the Barnes maze, and in impaired attentional capabilities in the Attentional set-shifting task. In the second part of the study, we did not observe any difference between experimental groups. We hypothesize that the experimental procedures utilized for the exogenous supplementation may have impacted our ability to observe the cognitive read-out *in vivo*. This study suggests that early life dietary sialylated HMOs play a crucial role in the development of cognitive functions. Future studies are needed to clarify if an exogenous supplementation of these oligosaccharides may compensate for these affected phenotypes.

## 1. Introduction

Human milk is the optimal source of nutrition for the newborn. Its composition results from an evolutionary adaptive biological process which, within the boundaries of physiological and environmental constraints, resulted in a nutritional source capable of preparing the newborn to the challenges to be encountered in future life. Breastfeeding is associated with multiple benefits for the infant and the mother herself: specifically, it exerts profound consequences on the development of the immune function and the gastrointestinal tract of the offspring (Fuhrer et al., 2010; ten Bruggencate et al., 2014) and favors protection against breast and ovarian cancer, and diabetes in the mother (Global Breastfeeding Collective, 2021a,b). These findings have been obtained in studies comparing breastfed (BF) and formula-fed (FF) infants. Additionally, maternal milk’s constituents persistently regulate the development of cognitive functions. In particular, experimental evidence indicates that breastfed infants show significantly higher intelligence scores compared to formula-fed children (Horwood et al., 2001; Victora et al., 2015).

Among the various components of human milk, sialic acid (Sia) has been proposed to exert a pivotal role whereby it acts as a constituent molecule of gangliosides and polySia-NCAM, both highly represented in the CNS and directly involved in neurogenesis, synaptogenesis, and the formation of neural circuits (Wang, 2012). The role exerted by Sia on brain patterning during the early post-natal stages has been proposed to contribute to the neurological and intellectual advantages conferred by breast- over formula-feeding (Wang et al., 2003; Wang, 2009; Schnaar et al., 2014). Specifically, human milk represents an essential source of Sia [in the form of sialylated human milk oligosaccharides (HMOs)] whereby the enzyme responsible for the endogenous synthesis of Sia has very low activity in the newborn (Martinez-Duncker et al., 2011). Human milk oligosaccharides (HMOs) represent about 8% of the milk oligosaccharides and have a wide range of beneficial effects on the physiology of the infant, including but not limited to supporting brain maturation. Preclinical studies indicate that their supplementation may beget substantial cognitive benefits (Morgan et al., 1981). Importantly, while HMOs are abundant in human milk, they have only recently been included into infant formula with limited quantity and diversity compared to human milk (ten Bruggencate et al., 2014).

In the present study, we investigated the role exerted by two sialylated HMOs, sialyl(alpha2,3)lactose (3′SL) and sialyl(alpha2,6)lactose (6′SL), on the development of cognitive capabilities. The selection of these HMOs rests upon the evidence that, while 3′SL and 6′SL are abundant in maternal milk, they are not adequately provided in infant formula (ten Bruggencate et al., 2014). Furthermore, our previous findings indicate that 3′SL (Pisa et al., 2021) and 6′SL (Hauser et al., 2021) alone may exert beneficial effects on the development of cognitive capabilities, specifically memory and attention. Based on these considerations, we tested whether the concurrent lack (6′SL) or reduction (3′SL) of these oligosaccharides may induce impairments in memory and attentional capabilities. Then we aimed at assessing whether the neonatal compensatory supplementation of 3′SL and 6′SL may prevent the observed cognitive impairments.

To test our hypothesis, we leveraged a mouse model characterized by a constitutive reduction/deficiency of 3′SL and 6′SL [B6.129-*St3gal4*^tm1.1Jxm^ - *St6gal1*^tm2Jxm^, hereafter double knock out (dKO)]. Previous studies showed that maternal milk provided by dams carrying the *St3Gal4* mutation is characterized by a remarkable reduction in 3′SL (Fuhrer et al., 2010), while maternal milk provided by dams carrying the *St6Gal1* mutation is characterized by a lack of 6′SL (Fuhrer et al., 2010). To test whether the selective and time-specific (i.e., lactation period) removal of 3′SL and 6′SL resulted in cognitive impairments in adulthood, we designed a cross-fostering experimental procedure in which wild-type mice received milk from dKO dams. Subjects were then tested for cognitive capabilities in adulthood (experiment 1).

Then, in experiment 2, we aimed at assessing whether the exogenous administration of these oligosaccharides was capable of compensating for the cognitive deficits. We therefore used a similar experimental design, with independent groups of wild-type mice reared to either wild-type (WT) or dKO dams receiving oral administration of 3′SL and 6′SL. The supplementation procedure started from post-natal day 1 and lasted until offspring began to eat solid food (post-natal day 16). This experimental design allowed to assess the long-lasting effect of a time selective exogenous supplementation of the aforementioned oligosaccharides in mice that received milk with reduced concentrations of 3′SL and absence of 6′SL. To assess the potential effects of the supplementation procedure *per se*, we also evaluated the profile exhibited by WT offspring reared to WT or dKO dams receiving water with the same experimental procedure.

## 2. Materials and methods

### 2.1. Animals and rearing conditions

Adult WT and heterozygous (HZ) B6.129-*St6gal1*^tm2Jxm^/J and HZ B6.129-*St3gal4*^tm1.1Jxm^/J were purchased from a commercial breeder (the Jackson Laboratory, Bar Harbor, ME, USA). Then, homozygous knock-out (KO-ST3) B6.129-*St3gal4*^tm1.1Jxm^/^tm1.1Jxm^ and homozygous knock-out (KO-ST6) B6.129-*St6gal1*^tm2Jxm^/^tm2Jxm^ were derived from heterozygous *St3gal4* B6.129-*St3gal4*^tm1.1Jxm^/J and *St6gal1* B6.129-*St6gal1*^tm2Jxm^/J mice [as described in Pisa et al. (2021) and Hauser et al. (2021), respectively]. Mice were housed in same-sex groups of 2–3 animals, in type-1 polycarbonate cages (33.0 × 13.0 × 14.0 cm) equipped with sawdust bedding, an enrichment bag (Mucedola, Settimo Milanese, Italy), metal top and *ad libitum* water and food pellets (Mucedola, Settimo Milanese, Italy). Mice lived in a reversed 12-h light-dark cycle (lights on at 18:00) in a room with a temperature of 21 ± 1°C and relative humidity of 60 ± 10%. Then, breeding triads were formed by one male of one KO genotype and two females of the opposite KO genotype, to produce heterozygous animals. Then, breeding triads were formed by one male and two females with heterozygous genotypes. From this breeding, dKO B6.129-*St3gal4*^tm1.1Jxm^/*St6gal1*^tm2Jxm^ animals were born with an average of 1/8 from every dam. Then, breeding triads were formed by one male and two females with the same genotype, to generate experimental animals. To generate the experimental colony, we mated 36 WT and 36 dKO female mice with 18 WT and 18 dKO male mice, respectively. Out of this batch, 24 WT and 20 dKO subjects were pregnant and gave birth to viable offspring.

### 2.2. Genotyping procedure

To extract genomic DNA, 0.3–0.5 cm of mouse tail biopsies were incubated at 55°C overnight in a heat block with gentle agitation (300 rpm) in 0.25–0.5 ml of Lysis Buffer pH 8 (100 mM Tris–HCl, 0.5% v/v TWEEN^®^ 20, 0.5% v/v NP-40, pH adjustment with HCl) with 0.2 mg/ml Proteinase K (Thermo Fisher Scientific, Waltham, MA, United States). After lysis, Proteinase K inactivation was performed by incubating samples at 75°C for 20 min. For *St6gal1* screening, 2 μL of each lysate were used as template to perform a PCR reaction with GoTaq^®^ G2 Flexi DNA Polymerase (Promega, Madison, WI, United States) (0.65U GoTaq^®^ G2 Flexi DNA Polymerase, 1x GoTaq^®^ Flexi Buffer, 1.5 mM MgCl_2_ Solution, 0.125 mM each dNTP, 0.6 μM primers, molecular biology grade water up to the final volume of 25 μL) using primers 15318 (Fw) 5′-ACTGTGGGGCTAACCTTTGA-3′ and 15319 (Rev) 5′-TGCACCATGACTCAGCTTCT-3′, as per B6.129-*St6gal1*^tm2jxm^/J strain genotyping protocol (JAX, 2022b). Amplifications were carried out using the following cycling conditions: 2 min at 95°C followed by 35 cycles of 95°C for 30 s, 53°C for 30 s, and 72°C for 40 s. A final extension step was performed at 72°C for 5 min. For *St3gal4* screening, 2 μL of each lysate were used to perform a PCR reaction (1.3U GoTaq^®^ DNA Polymerase, 1x GoTaq^®^ Flexi Buffer, 1.5 mM MgCl_2_ Solution, 0.125 mM each dNTP, 0.6 μM primers, molecular biology grade water up to the final volume of 25 μL) using primers oIMR6890 (Fw) 5′-GACGCCATCCACCTATGAG-3′, oIMR6891 (Rev) 5′-GGCTGCTCCCATTCCACT-3′, oIMR6892 (Rev) 5′-GGCTCTTTGTGGGACCATCAG-3′ as per B6.129-*St3gal4*^tm1.1Jxm^/J strain genotyping protocol (JAX, 2022a). Amplifications were carried out using the following cycling conditions: 2 min at 95°C followed by 34 cycles of 95°C for 30 s, 66°C for 1 min, and 72°C for 40 s. A final extension step was performed at 72°C for 5 min. PCR reactions were kept at 4°C until being electrophoresed in 2% agarose 1 × TBE (89 mM Tris base, 89 mM boric acid and 2 mM ethylenediaminetetraacetic acid) gels for 1 h at 85 V. For visualization of electrophoresed PCR products, gels were stained with Ethidium bromide (0.006% v/v) and digital images were captured in a CHEMIDOC MP Imaging System (Bio-Rad, Hercules, CA, United States). Homozygous wild type, heterozygous mutant, and homozygous mutant genotypes have been distinguished by their different band patterns on gel, as reported in relative genotyping protocols (for *St6gal1*: 287 bp, 287 bp and 403 bp, 403 bp, respectively. For *St3Gal4*: 290 bp, 290 bp and 450 bp, 450 bp, respectively).

### 2.3. Fostering procedure, supplementation procedure, and rearing

**FIGURE 1:**
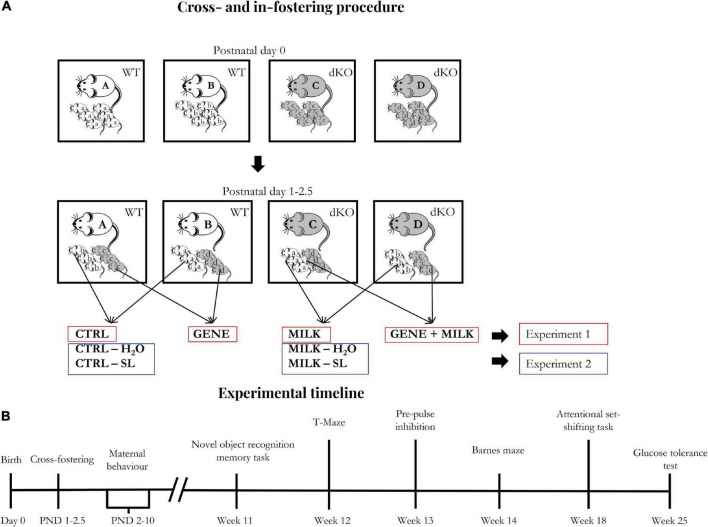
**(A)** Fostering scheme: Starting one day after birth, cross- and in-fostering procedures were performed as sketched in the lower panels. Dams remained in their home cages while offspring were transferred from their original cages to those housing their foster dams. At the end of fostering procedures, litters consisted of WT and dKO mice in a 1:1 ratio. Importantly, no pup was reared to its biological dam. **(B)** Experimental timeline: The day of birth was designated as day 0. The experimental procedures started between post-natal days (PND) 1 and 2.5 (cross- and in-fostering procedures).

As performed in our previous studies (Hauser et al., 2021; Pisa et al., 2021), cross-fostering procedures were executed 24–60 h after birth to minimize the number of animals to be discarded due to the absence of foster dams, using four dams (two dKO and two WT) at the same time (see Figure 1A). The day of birth was designated as post-natal day 0 (PND 0). On the day of fostering (PND 1–2.5), dams were removed and placed in a clean cage during the time of the procedure. Pups were first sexed, then marked through toe tattoo ink puncture (Ketchum Manufacturing Inc., Brockville, ON, Canada) (Chen et al., 2016). After the marking procedure, pups were moved to the cage containing the foster dam and covered with sawdust. Regarding experiment 1, each dam received a mixed litter composed of WT and dKO male and female offspring with a 1:1 ratio among all variables. This ratio was not attained in four out of 22 instances, in which the ratio was 2 WT:1dKO. Importantly, all mice were reared to foster dams. At weaning (PND 28), male mice were ear-marked and then housed in type-1 polycarbonate cages, in groups of 2–3 animals (belonging to different experimental groups). At the end of fostering and weaning procedures we obtained the following experimental groups:

- N = 13 WT offspring reared to WT dams (Control, hereafter CTRL group)
- N = 12 WT offspring reared to dKO dams (MILK group)
- N = 12 dKO offspring reared to WT dams (GENE group)
- N = 9 dKO offspring reared to dKO dams (GENE + MILK group)

Regarding experiment 2, one day after fostering (PND 2–3.5), we started the supplementation procedure which lasted until PND 16. The supplementation procedure was performed only in WT subjects, both reared to WT or dKO dams. The supplementation procedure was performed in two daily sessions entailing a 30 min maternal separation followed by a 15 s supplementation procedure. Each pup was individually administered with an amount of 3′SL and 6′SL roughly equivalent to those reported in mouse milk, as follows: after having hand-picked a pup, the operator used a mechanic pipette (PIPETMAN, Classic, Gilson, Middleton, WI, USA) to introduce water or 3′SL–6′SL solution in the mouth of the pups (i.e., 3 μL of solution). Specifically, the pipette was gently placed on the lips of the pups until they suckled the solution. To calculate the amount of 3′SL and 6′SL, we rested upon the daily intake of milk in pups between PND 1–16 (Barnett and Dickson, 1984; Hoshiba, 2004) and upon the known concentrations of 3′SL and 6′SL in milk during this period (Fuhrer et al., 2010). The exact concentrations of 3′SL and 6′SL throughout the administration period are reported in Table 1.

**TABLE 1:** Concentrations of 3′SL and 6′SL between PND 1 and PND 16 expressed in mg/g of body weight (BW) of the mice.

HMOs	PND 1	PND 2–6	PND 7–12	PND 13–16
3′SL	1.36 mg/g of BW	1.95 mg/g of BW	1.49 mg/g of BW	0.32 mg/g of BW
6′SL	0.48 mg/g of BW	0.92 mg/g of BW	0.92 mg/g of BW	0.6 mg/g of BW

The solution of 3′SL and 6′SL (expressed in mg/g of body weight) was prepared daily by dissolving these in tap water at room temperature. Specifically, we prepared the solution progressively adding to the water 3′SL and 6′SL into a glass baker on a magnetic stir plate. Then, it was agitated for 10 min until the solution was clear. After the supplementation procedure, dams were then relocated in their home cage with the pups. At weaning (PND 28), male mice were ear-marked and then housed in type-1 polycarbonate cages, in groups of 2–3 animals (belonging to different experimental groups). These fostering, supplementation and weaning procedures resulted in the following experimental groups:

- N = 12 WT offspring reared to WT dams and receiving water (CTRL - H_2_O group)
- N = 12 WT offspring reared to dKO dams and receiving water (MILK - H_2_O group)
- N = 12 WT offspring reared to WT dams and receiving 3′SL–6′SL solution (CTRL - SL group)
- N = 12 WT offspring reared to dKO dams and receiving 3′SL–6′SL solution (MILK - SL group).

In both experiments 1 and 2, in order to minimize the number of subjects used in each experiment, we adopted a split-litter cross-fostering design. Thus, each litter was composed of an identical number of WT and dKO offspring. The aforementioned subjects have then been evaluated for cognitive capabilities (attention and memory performances) and metabolic responses (glucose tolerance) through the test battery outlined in Figure 1B.

### 2.4. Behavioral assessment

Behavioral testing was performed in an air-conditioned room (temperature 21 ± 1°C and relative humidity 60 ± 10%) adjacent to the housing room. Whilst Barnes maze test was conducted under bright light (85 lux), all other tests were conducted under dim light (10 lux). With respect to behavioral outcomes, Pre-pulse inhibition (PPI), and Barnes maze were quantified by automated software (SOF-815, med associates Inc., St Albans, VT, United States of America for PPI and “The EthoVision,” Noldus, Wageningen, the Netherlands, for Barnes maze) which by definition is blind to treatments. Behavioral outcomes of Novel Object Recognition (NOR) task were quantified by an operator blinded to the allocation of the experimental group through a tracking software (“The Observer XT,” Noldus, Wageningen, the Netherlands). Then, for T-maze and glucose tolerance test, the experimenter conducting the test received the mouse from another experimenter who guaranteed blinding. For the attentional set-shifting task and maternal behavior, test blinding was not possible.

#### 2.4.1. Maternal behavior

After the cross-fostering procedure, we analyzed maternal behavior in 10 WT and seven dKO dams between PND 1 and 10. Maternal care was observed once every 2 days for two 1-h sessions during the dark phase. Each session lasted 1 h in which we scored 20 instantaneous samples (interspaced by 3-min intervals) for each dam. Herein, we assessed absolute levels of active maternal care defined as high kyphosis, low-partial kyphosis, licking and grooming [see Macrì et al. (2007) for details]. Data represented are frequency/h.

#### 2.4.2. T-maze

We analyzed spontaneous alternation through the T-maze test. The apparatus is a T-shaped maze, in which three equally sized arms are enclosed by protective walls (14.5 cm × 8 cm; Technosmart Europe srl, Rome, Italy). Two experimental sessions per day were performed on five consecutive days. Each session consisted of two trials, starting with the mouse in the start compartment facing the wall. The animal was allowed to explore the apparatus for 2 min. A trial was considered complete when the mouse entered one of the two alternative arms; this was scored as the first choice and the arm door was closed. After a few seconds, the subject was positioned again in the start compartment and the second trial was performed. If the mouse entered the arm opposite to the one chosen in the first trial, an alternation was scored. Alternation percentage (the number of alternations divided by the number of completed sessions × 100) was scored for each animal. Before each trial, the apparatus was cleaned with a 30% ethanol/water solution.

#### 2.4.3. Novel object recognition task

To evaluate recognition memory, mice were tested individually in a NOR Test, into a dark familiar arena (40 cm × 40 cm × 40 cm, Technosmart Europe Srl, Rome, Italy) equipped with a camera (Sony Handycam DCR-SX21E, Tokyo, Japan) under indirect dim light (10 lux). NOR protocol includes a 10 min familiarization phase and a 10 min test trial conducted 24 h after the familiarization phase. Twenty-four hours before familiarization mice were allowed to explore the empty apparatus for 30 min (habituation phase). Two types of plastic objects (A and B) of similar volume and different shapes were used as stimuli in test. We used six pairs of objects A and six pairs of objects B. The use of each set of objects was counterbalanced between experimental groups so that each object was used either as a familiar object or as a novel object an equal number of times. During familiarization, two identical unfamiliar plastic objects (e.g., A + A) were located inside the arena, 9 cm away from the walls and 18 cm from each other. The test trial was performed 24 h after the familiarization phase: one familiar (e.g., A) object and a new unfamiliar (e.g., B) object were placed inside the arena, in the same position as familiarization phase objects. Before each phase, the apparatus and the objects were cleaned with a 30% ethanol/water solution. Each phase was recorded by a video-camera and data were scored offline using dedicated software (The Observer XT 10; Noldus, The Netherlands) by a single operator (intra-rater reliability coefficient 0.99). Interaction with an object was scored when the subject touched it with the nose and/or paws or directed the nose toward an object at less than 1 cm. NOR memory was measured with the discrimination index (DI) as the difference between the time of interaction with the new and familiar object divided by the total amount of time of interaction with the two objects (Antunes and Biala, 2012).

#### 2.4.4. Barnes maze

To analyze spatial memory, mice were tested using a Barnes Maze Task. In this experiment, mice, exposed to a bright light (85 lux) on a circular white arena (diameter 92 cm) elevated 93 cm above the floor, have to locate an escape box (7 cm × 37 cm × 9 cm) placed underneath one of 20 holes (target hole, diameter 5 cm, Technosmart Europe srl, Rome, Italy), while the other 19 holes are covered with a cap providing the same visual cue as the target hole. Experiments were conducted in an experimental room adjacent to the housing room. The experimental protocol consisted of one day of habituation, 4 days of training and two probe trials: the latter were conducted 24 h or 7 days after the last training session. The position of the escape box was constant within individuals and randomized between mice. Habituation consisted of two consecutive trials in which the subject was allowed to explore the apparatus. The trial ended when the animal located the escape box, or after 60 s of exploration when an operator gently directed the mouse to the escape hole. After each trial, mice were left inside the escape box for 2 min. During training (days 2–5), animals were exposed daily to two consecutive trials interspaced by a 10-min interval. The trial ended when the animal located the escape box, or after 3 min when an operator gently directed the mouse to the right hole. Once the mouse was in the escape box, it was left there for 1 min, before being returned to the home cage. The probe trial consisted of a 90-s free exploration during which the escape box was removed, and the holes were covered with the cups. Each trial was recorded by a video camera and analyzed through a tracking software (“The EthoVision,” Noldus, The Netherlands) which provides information on the path traveled, time spent in different zones, and latency to reach the target hole. Spatial memory was evaluated through the time spent in the target hole zone during the probe trials. Between every trial, the apparatus and the escape box were cleaned with a 30% ethanol/water solution.

#### 2.4.5. Pre-pulse inhibition

##### 2.4.5.1. Apparatus

The apparatus is constituted by a foam-lined isolation chamber (ENV-018S, Med Associates Inc., St Albans, VT, United States of America), which presents an acoustic stimulator (ANL-925, Med Associates Inc., St Albans, VT, United States of America) and a platform with a transducer amplifier (PHM-250-60, Med Associates Inc., St Albans, VT, United States of America). Inside the chamber, red light and a fan guaranteed dimmer light and ventilation. The mouse was placed in a perforated compartment above the platform. Data were recorded through dedicated software (SOF-815, Med Associates Inc., St Albans, VT, United States of America).

##### 2.4.5.2. Experimental procedure

Twenty-four hours before testing, mice were habituated individually to the startle chamber for 5 min with a fan and the red light turned on. The testing phase consisted of an acclimation phase and three consecutive blocks. During the acclimation phase, mice, placed inside the startle chamber, were exposed to white noise (62 dB) for 5 min. The first block consisted of ten pulse alone trials (120 dB pulses), with an average 15-s inter-trial interval. Then the second block started. This session lasted 16 min and consisted of 56 trials comprising four types of trials presented in a pseudorandomized order, with a variable inter-trial interval (10 or 20 s), to avoid habituation. Trials proposed were: pre-pulse alone – two for each pre-pulse intensity (74, 78, 82, or 84 dB) –, pre-pulse plus pulse – eight trials for pre-pulse intensity – eight trials of pulse alone, and eight no stimulation trials. Each trial started with a 50 ms null period. Then a pre-pulse, followed by a startle stimulus (white noise 40 ms long, with an intensity of 120 dB) were presented. The inter-stimulus period was 100 ms long.

The galvanic response was evaluated 65 ms after the onset of the startle. Pre-pulse inhibition was computed as the percentage of reduction of startle to the pre-pulse + pulse trials compared to the pulse alone trial: 1 − (startle_pre–pulse+pulse_/startle_pulse_ _alone_). Between every animal, the apparatus was cleaned with a 30% ethanol/water solution.

#### 2.4.6. Attentional set-shifting task

We adopted the attentional set-shifting task, developed by Birrell and Brown (2000) and modified by Colacicco et al. (2002) and Macrì et al. (2009).

##### 2.4.6.1. Apparatus

The apparatus was an opaque PVC U-shaped box, with a grid floor (40 cm × 30 cm × 15 cm). It was composed of two identical choice compartments (15 cm × 15 cm), which could be reached by the experimental subject from the starting compartment (30 cm × 30 cm). In each choice compartment, we placed a cylindrical cup (40 mm diameter, 35 mm high), baited with a quarter of cereal (35 mg, Honey Cheerios, Nestlé), covered with a scented digging medium. Tactile or olfactory stimuli indicated the presence or absence of reward in a cup.

##### 2.4.6.2. Experimental procedure

Three days before the beginning of the experiment, mice were food restricted to improve their motivation to perform the task, maintaining them at 95% of their *ad libitum* body weight (we allowed 3 h of *ad libitum* access to food each day). Habituation and training phases were conducted 1 day before testing. During habituation, mice were allowed to explore the apparatus for 10 min. After habituation, we conducted the training phase: a sequence of nine consecutive trials to train the subject to dig into bowls to find the food reward. During the first three trials, the reward was placed on the surface of both empty cups. During trials 3–6 food rewards were on the surface of the digging medium inside the cups. Between trials 6–9 the food reward was located underneath the digging medium. Animals were allowed to pass to the subsequent trial if they ate both rewards. The test phase is composed of five consecutive stages: simple discrimination (SD) in which mice had to discriminate between two stimuli of the same dimension (e.g., odor, thyme vs. cinnamon); compound discrimination (CD) in which mice had to perform the same discrimination while a second, confounding, dimension was introduced. For example, the digging medium was varied while the odor remained the relevant stimulus; reversal of compound discrimination (CDR) in which everything was unchanged, but the wrong choice became the right one (e.g., food reward was no longer associated with cinnamon but with thyme); in intra-dimensional shift we changed all the stimuli, but odor remained the relevant dimension, while in extra-dimensional shift digging medium became the food rewarded associated stimulus (see Table 2). Each stage consisted of a series of consecutive trials which started when the animal was placed inside the starting compartment and the sliding doors were opened. The first cup where the mouse dug was scored. The side and order of presentation of each dimension were randomized. During the first four trials (free trial) of a new stage, mice who committed an error, which was, however, scored, was left free to explore the apparatus until it found the food reward in the correct cup. After the correct choice, the mouse was allowed to eat the cereal found and then placed back in the starting compartment. Mice reached the learning criterion when they performed 8 correct choices out of 10 consecutive trials. Between each experimental subject, the apparatus was cleaned with a 30% ethanol/water solution.

**TABLE 2:** Stimulus exemplars used in the Attentional set-shifting task (ASST).

Stage	Relevant dimension	Rewarded stimulus	Discrimination 1	Discrimination 2
SD	Olfactory	Cinnamon	Cinnamon vs. sage (in bedding material)	
CD	Olfactory	Cinnamon	Sawdust + cinnamon vs. cotton + sage	Sawdust + sage vs. cotton + cinnamon
CDr	Olfactory	Sage	Sawdust + cinnamon vs. cotton + sage	Sawdust + sage vs. cotton + cinnamon
IDs	Olfactory	Ginger	Crepe paper + ginger vs. confetti + coriander	Crepe paper + coriander vs. confetti + ginger
EDs	Tactile-visual	Polystyrene	Polystyrene + cloves vs. tissue paper + rosemary	Polystyrene + rosemary vs. tissue paper + cloves

Stimuli used in the testing phase (first column), relevant dimension (second column), and rewarded stimulus (third column). In each stage, mice were presented with either Discrimination 1 (fourth column) or Discrimination 2 (fifth columns), according to a pseudo-random sequence. The side where each pair of stimuli was presented (left and right bowls) were counterbalanced through a pseudo-random sequence. The entire procedure lasted 5 days.

#### 2.4.7. Glucose tolerance test

Blood glucose concentrations were measured through a commercial glucometer (Accu-Chek Active, Roche Diagnostics) before and after an intraperitoneal (IP) injection of 2 g/kg body weight d-glucose (10% d-glucose solution; Sigma, St. Louis, MO, USA). Specifically, the mice were food-deprived for 15 h (18.30–9.30) and then injected IP with glucose. Blood glucose concentration was measured in baseline conditions (before the injection, t0), and then 20, 40, 60, 120 min after glucose injection. Peripheral blood samples were obtained from the central part of the tail, by the tail nick procedure (Fluttert et al., 2000), performed through a commercial razor for callus removers (SOLINGEN^®^, Solingen, Germany). To account for the integral response to glucose administration, we also calculated the area under the curve using the trapezoidal rule.

### 2.5. Statistical analyses

All statistical analyses were performed using StatView. Analysis of variance (ANOVA) was used for split-plot design. The general model for the first study implied two offspring genotypes (WT vs. dKO) × two maternal genotypes (WT vs. dKO) × k (repeated measurements, variable depending on the specific test). Offspring and maternal genotype were between-subjects factors, and k was the within-subjects factor. For the second study, the model implied two maternal genotypes (WT vs. dKO) × two treatments (H_2_O vs. SL) × k (repeated measurements, variable depending on the specific test). Maternal genotype and treatment were between-subjects factors, and k was the within-subjects factor. Tukey HSD *post-hoc* tests were used when allowed. Data were expressed as mean ± SEM. Statistical significance was set at α < 0.05. In experiments in which a given threshold was required to evaluate if the animal met the criterion for the experimental paradigm, we compared the performance to the threshold through confidence interval (CI), which was used to evaluate whether the criterion was achieved or not.

## 3. Results

### 3.1. Maternal behavior

We observed that absolute levels of active maternal care steadily declined in all experimental subjects between PND 1 and 10 (day: F_4_,_60_ = 7.22 *p*-value < 0.0001). WT and dKO dams provided an indistinguishable level of active maternal care to their offspring (maternal genotype: F_1_,_15_ = 0.15 *p*-value = 0.7; interaction between day and maternal genotype: F_4_,_60_ = 2.37 *p*-value = 0.063, see Supplementary Figure 1).

### 3.2. T-maze

**FIGURE 2:**
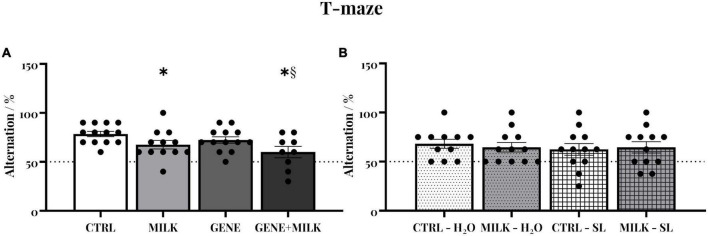
Spontaneous alternation: Percentage of alternations between the two arms of the T-maze in experiment 1 **(A)** and experiment 2 **(B)**. The dashed lines represent the chance level. *p < 0.05 in *post-hoc* tests compared to CTRL group, §*p* < 0.05 in *post-hoc* tests compared to GENE group.

Control (CTRL) subjects of experiment 1 exhibited a natural tendency to alternate between the two arms of the maze (average 78.46%; 95% CI 73.58–83.34). This tendency varied depending on the maternal genotype, with MILK and GENE + MILK groups exhibiting reduced spontaneous alternation compared to CTRL and GENE groups, respectively (maternal genotype: F_1_,_43_ = 8.77 *p*-value = 0.005). There was no impact of the offspring genotype (offspring genotype: F_1_,_43_ = 3.02 *p*-value = 0.09). There was, however, a significant interaction between offspring and maternal genotype (F_1_,_43_ = 0.03 *p*-value = 0.86; *p* < 0.05 in *post-hoc* tests), whereby the GENE + MILK group exhibited the lowest level of spontaneous alternation compared to all other groups (see Figure 2A and Supplementary Figure 2). Regarding experiment 2, CTRL subjects exhibited an intact natural tendency to alternate between the two arms of the maze (95% CI 59.97–79.38). There was no significant difference in the percentage of alternations between groups (maternal genotype: F_1_,_43_ = 0.03 *p*-value = 0.89; treatment: F_1_,_43_ = 0.28 *p*-value = 0.6; interaction between maternal genotype and treatment: F_1_,_43_ = 0.28 *p*-value = 0.6, see Figure 2B).

### 3.3. Novel object recognition task

**FIGURE 3:**
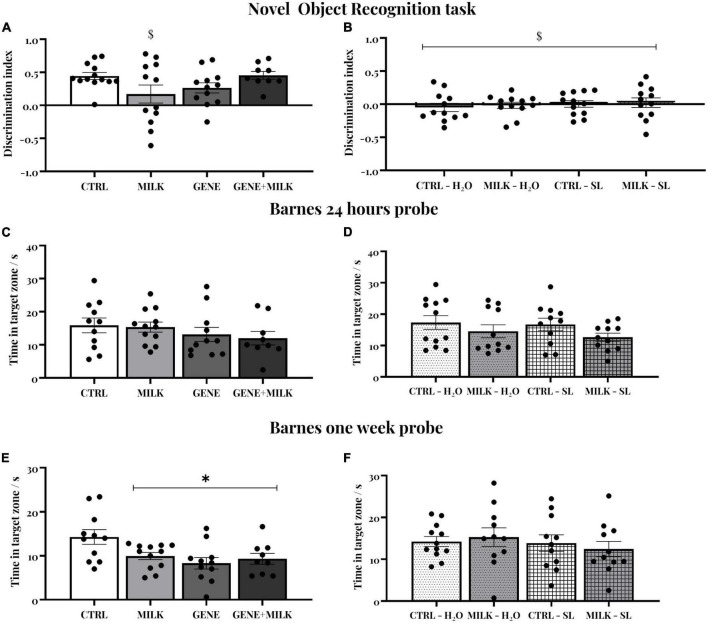
**(A,B)** Novel object recognition (NOR) test: Discrimination index for the novel object during 10-min test sessions performed 24 h after the familiarization phase in experiment 1 **(A)** and experiment 2 **(B)**. The dashed lines represent the chance level. $ indicate that experimental groups did not differ from the chance level. **(C,D)** Short-term spatial memory: time spent in target zone during the probe trial conducted 24 h after the last day of training in the experiment 1 **(C)** and experiment 2 **(D)**. **(E,F)** Long-term spatial memory: time spent in the target zone during the probe trial conducted 7 days after the last day of training. In experiment 1 **(E)**, all groups showed reduced long-term spatial memory compared to the CTRL group. *p < 0.05 in *post-hoc* tests compared to CTRL group.

In experiment 1, we observed an overall preference for the novel over the familiar object in all experimental subjects. This finding was supported by a DI significantly different from chance (average DI = 0.329 ± 0.064). Although the main effects of offspring genotype (F_1_,_42_ = 0.32 *p*-value = 0.57) and maternal genotype (F_1_,_42_ = 0.22 *p*-value = 0.64) apparently suggested the absence of differences between experimental groups, we observed that the maternal genotype acted differently depending on the offspring genotype (interaction between maternal genotype and offspring genotype: F_1_,_42_ = 6.44 *p*-value = 0.015); specifically, while CTRL, GENE, and GENE + MILK mice exhibited a significant preference for the novel object, MILK mice failed to show such preference (Figure 3A). Finally, we observed that the absolute time spent exploring both objects did not differ between experimental groups (see Supplementary Table 1). While a similar absence of difference in absolute time spent exploring the objects was also observed in experiment 2 (see Supplementary Table 2), in this experiment we also observed that all experimental subjects failed to show any preference for the novel over the familiar object (average DI = −0.009 ± 0.040, and Figure 3B).

### 3.4. Barnes maze

Spatial memory was evaluated through a Barnes Maze test. All subjects acquired the position of the escape box during the acquisition without any difference between groups in their latency to reach the escape box in experiment 1 (day: F_5_,_195_ = 25.21 *p*-value ≤ 0.0001; offspring genotype: F_1_,_39_ = 0.41 *p*-value = 0.53; interaction between day and offspring genotype: F_5_,_195_ = 1.88 *p*-value = 0.099; maternal genotype: F_1_,_39_ = 2.28 *p*-value = 0.14; interaction between day and maternal genotype: F_5_,_195_ = 1.13 *p*-value = 0.35; interaction between offspring and maternal genotype: F_1_,_39_ = 0.11 *p*-value = 0.14; interaction between day, offspring and maternal genotype: F_5_,_195_ = 0.76 *p*-value = 0.57). Then, we measured time spent in the target zone in two probe trials conducted 24 h (short-term memory, Figure 3C) and one week (long-term memory, Figure 3E and Supplementary Figure 3) after the last training session. There was no difference between groups in short-term memory retention (offspring genotype: F_1_,_39_ = 2.33 *p*-value = 0.13; maternal genotype: F_1_,_39_ = 0.17 *p*-value = 0.68; interaction between offspring and maternal genotype: F_1_,_39_ = 0.02 *p*-value = 0.88). One week later, long-term spatial memory varied between experimental groups: specifically, all experimental groups exhibited reduced long-term spatial memory compared to CTRL group (offspring genotype: F_1_,_39_ = 6.46 *p*-value = 0.01; maternal genotype: F_1_,_39_ = 1.62 *p*-value = 0.21; interaction between offspring and maternal genotype: F_1_,_39_ = 4.25 *p*-value = 0.04; *p* < 0.05 in *post-hoc* tests between control and all three other experimental groups). Regarding experiment 2, there was no difference between groups during acquisition (day: F_5_,_205_ = 19.202 *p*-value ≤ 0.0001; maternal genotype: F_1_,_41_ = 0.36 *p*-value = 0.54; interaction between day and maternal genotype: F_5_,_205_ = 0.383 *p*-value = 0.86; treatment: F_1_,_41_ = 1.84 *p*-value = 0.18; interaction between day and treatment: F_5_,_205_ = 0.55 *p*-value = 0.74; interaction between maternal genotype and treatment: F_1_,_41_ = 1.71 *p*-value = 0.2; interaction between day, maternal genotype and treatment: F_5_,_205_ = 0.25 *p*-value = 0.94) nor in the short- and long-term memory probes (maternal genotype: F_1_,_41_ = 3.13 *p*-value = 0.08; treatment: F_1_,_41_ = 0.42 *p*-value = 0.52; interaction between maternal genotype and treatment: F_1_,_41_ = 0.11 *p*-value = 0.74 for short term memory retention, see Figure 3D; maternal genotype: F_1_,_41_ = 0.012 *p*-value = 0.91; treatment: F_1_,_41_ = 0.78 *p*-value = 0.38; interaction between maternal genotype and treatment: F_1_,_41_ = 0.46 *p*-value = 0.50 for long term memory retention, see Figure 3F).

### 3.5. Pre-pulse inhibition

**FIGURE 4:**
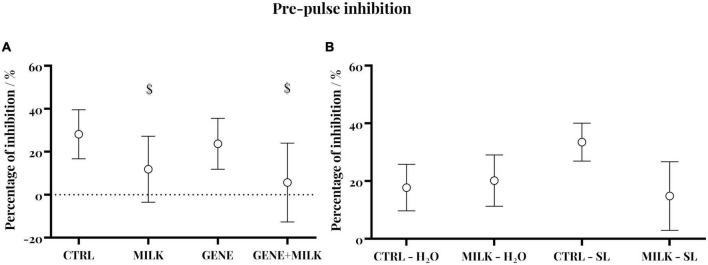
Pre-pulse inhibition in experiment 1 **(A)** and experiment 2 **(B)**: Percent inhibition of the startle reflex measured as PPI = [(A–B)/A × 100]: In panel **(A)** is the Galvanic reflex in response to the startle stimulus alone, and in panel **(B)** is the response to pre-pulse plus pulse stimuli (circular symbol) with 95% CI (whiskers). $ indicate that experimental groups did not differ from the chance level.

Regarding experiment 1, CTRL subjects exhibited intact PPI as shown by the reduced startle in response to pre-pulse plus pulse stimuli compared to pulse-alone trials (95% CI 12.59 to 22.55). Yet, this capability varied depending on the maternal genotype whereby, regardless of their genotype, adult offspring reared to KO dams failed to show intact PPI (95% CI −0.5–23.01; −9.13–20.45 for MILK and MILK + GENE, respectively). Conversely, GENE offspring showed an intact PPI (95% CI 12.12–33.93 and 10.97–30.05, respectively), Figure 4A. For experiment 2, all groups exhibited an intact pre-pulse inhibition, with no difference between experimental groups, see Figure 4B.

### 3.6. Attentional set-shifting task

**FIGURE 5:**
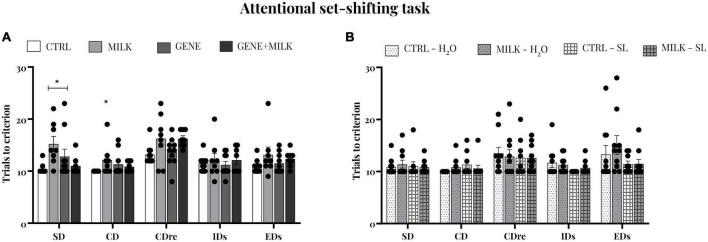
Attentional set-shifting task, trials to reach criterion: number of trials completed to attain the criterion of all phases in experiment 1 **(A)** and experiment 2 **(B)**. Regarding the first study, the MILK group committed required a higher number of trials to criterion compared to CTRL group; *p < 0.05 in *post-hoc* tests compared to CTRL group.

To assess whether experimental subjects differed in cognitive flexibility and attentional capabilities, we performed the attentional set-shifting task. In the experiment 1, during the SD phase, MILK subjects required more trials to obtain the criterion of CTRL group (offspring genotype: F_1_,_32_ = 0.75 *p*-value = 0.39; maternal genotype: F_1_,_32_ = 2.03 *p*-value = 0.16; interaction between offspring and maternal genotype: F_1_,_32_ = 10.1 *p*-value = 0.0039, *p* < 0.05 in *post-hoc* tests). In CD phase, MILK subjects required again more trials during the stage (offspring genotype: F_1_,_32_ = 0.03 *p*-value = 0.97; maternal genotype: F_1_,_32_ = 1.74 *p*-value = 0.20; interaction between offspring and maternal genotype: F_1_,_32_ = 3.9 *p*-value = 0.06, *p* < 0.05 in *post-hoc* tests; data on errors to criterion are reported in the Supplementary material, see Supplementary Figure 4A). During CDR, IDs, and EDs phases, there was no difference between group in trials and errors (data not shown, see Figure 5A and Supplementary Figure 4A). Albeit these results, in the experiment 2 we did not observe any differences between groups during all the phases of the test, both in trials and in errors (see Figure 5B and Supplementary Figure 4B).

### 3.7. Glucose tolerance

**FIGURE 6:**
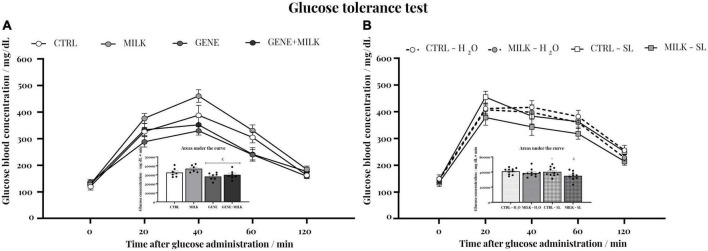
Glucose tolerance test: blood glucose concentrations before and after a glucose (2 g/kg body weight) injection in experiment 1 **(A)** and experiment 2 **(B)**; inset: integral response to glucose administration plotted as the area under the curve. €*p* < 0.05 in *post-hoc* tests compared MILK group. × *p* < 0.05 in *post-hoc* tests compared to CTRL – H_2_O group; +*p* < 0.05 in *post-hoc* tests compared to MILK – SL group.

In both studies, each experimental group exhibited the expected response to the glucose administration (2 g/kg body weight): blood glucose concentration raised rapidly, reaching the maximal value 20 min after the administration, and then steadily declined to attain basal concentrations 120 min after the injection. In the first study, this response varied depending on offspring genotype, with KO mice showing lower response to glucose compared to WT mice (time: F_4_,_92_ = 187.19 *p*-value ≤ 0.0001; offspring genotype: F_1_,_23_ = 9.65 *p*-value = 0.005; interaction between time and offspring genotype: F_4_,_92_ = 6.85 *p*-value ≤ 0.0001; maternal genotype: F_1_,_23_ = 3.95 *p*-value = 0.06; interaction between time and maternal genotype: F_4_,_92_ = 1.83 *p*-value = 0.13; interaction between offspring and maternal genotype: F_1_,_23_ = 0.59 *p*-value = 0.45; interaction between time, offspring and maternal genotype: F_4_,_92_ = 0.35 *p*-value = 0.84). Specifically, when assessing the area under the curve, the GENE group exhibited a reduced response compared to the MILK group (*p* < 0.05 in *post-hoc* tests, see Figure 6A and Supplementary Figure 5). In experiment 2, mice reared with a milk without 3′SL and 6′SL but receiving exogenously the oligosaccharides, exhibited a lower reactivity to the injection of glucose compared to CTRL subjects (time: F_4_,_144_ = 155.09 *p*-value ≤ 0.0001; maternal genotype: F_1_,_36_ = 5.4 *p*-value = 0.026; interaction between time and maternal genotype: F_4_,_144_ = 0.52 *p*-value = 0.72; treatment: F_1_,_36_ = 1.96 *p*-value = 0.17; interaction between time and treatment: F_4_,_144_ = 1.22 *p*-value = 0.3 interaction between maternal genotype and treatment: F_1_,_36_ = 1.17 *p*-value = 0.29; interaction between time, maternal genotype and treatment: F_4_,_144_ = 0.53 *p*-value = 0.71 *p* < 0.05 in *post-hoc* tests, see Figure 6B and Supplementary Figure 6).

### 3.8. Body weight

**FIGURE 7:**
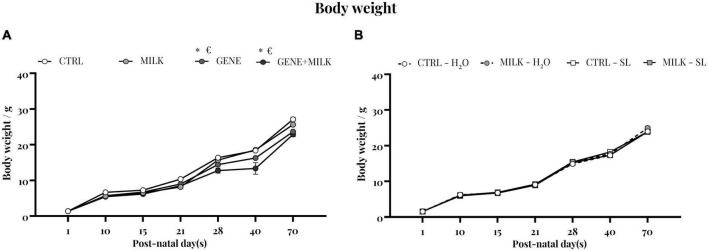
Body weight: body weight measurement of experiment 1 **(A)** and experiment 2 **(B)**. *p < 0.05 in *post-hoc* tests compared to CTRL group, €*p* < 0.05 in *post-hoc* tests compared to GENE group.

Body weight was measured during early stage of life and until the beginning of the experimental test battery. In the first study, the weight varied depending on the maternal genotype, with KO subjects showing reduced body weight compared to WT mice (time: F_6_,_258_ = 2287.216 *p*-value ≤ 0.0001; offspring genotype: F_1_,_43_ = 38.88 *p*-value ≤ 0.0001; interaction between time and offspring genotype: F_6_,_258_ = 19.9 *p*-value ≤ 0.0001; maternal genotype: F_1_,_43_ = 13.62 *p*-value = 0.0006; interaction between time and maternal genotype: F_6_,_258_ = 2.32 *p*-value = 0.03; interaction between offspring and maternal genotype: F_1_,_43_ = 0.063 *p*-value = 0.8; interaction between time, offspring and maternal genotype: F_6_,_258_ = 5.51 *p*-value ≤ 0.0001, see Figure 7A). In experiment 2 there was no difference between experimental groups (time: F_6_,_264_ = 6816.52 *p*-value ≤ 0.0001; maternal genotype: F_1_,_44_ = 0.05 *p*-value = 0.83; interaction between time and maternal genotype: F_6_,_264_ = 1.7 *p*-value = 0.12; treatment: F_1_,_44_ = 0.38 *p*-value = 0.54; interaction between time and treatment: F_6_,_264_ = 1.91 *p*-value = 0.07; interaction between maternal genotype and treatment: F_1_,_44_ = 12.5 *p*-value = 0.001; interaction between time, maternal genotype and treatment: F_6_,_264_ = 1.93 *p*-value = 0.07 see Figure 7B).

## 4. Discussion

In the present study, we observed that the concurrent reduction of 3′SL and absence of 6′SL during lactation resulted in an impairment in memory and attentional capabilities. Yet, when we investigated whether an exogenous oral supplementation of these oligosaccharides could contrast the observed deficits, we failed to observe remarkable differences between the experimental groups. This indistinguishable between-group phenotype also extended to mice receiving vehicle, thus suggesting that the experimental manipulation necessary to supplement 3′SL and 6′SL likely abolished interindividual differences. This ultimately resulted in a reduced possibility to detect any potential compensatory effect of the exogenous supplementation itself.

### 4.1. Selective deprivation of 3′SL and 6′SL impaired memory and attention

Our data show that WT mice reared with a reduction of 3′SL and an absence of 6′SL exhibited, in adulthood, reduced spatial, working and recognition memory compared to WT mice receiving normal milk. These findings confirm our previous studies evaluating the effect of the lack of 6′SL and reduction of 3′SL alone on memory capabilities (Hauser et al., 2021; Pisa et al., 2021). Furthermore, this result is also in agreement with other studies that reported beneficial effects of dietary supplementation of sialic acid in piglets (Wang et al., 2007) and sialylated oligosaccharides in preterm pigs (Obelitz-Ryom et al., 2019) and mice (Tarr et al., 2015). Yet, the present work overcame several limitations of these aforementioned studies, which have already been discussed in Hauser et al. (2021) and Pisa et al. (2021). Our experimental design, which allowed us to limit the selective reduction of 3′SL and deprivation of 6′SL to the lactation period, suggests that the selective absence of these oligosaccharides during the limited time window of lactation may consistently impair different types of memory later in adulthood.

Furthermore, besides memory, we observed that WT mice reared in the absence of 3′SL and 6′SL exhibited reduced attentional capabilities compared to control mice. This result is also in line with our previous studies showing how reduced neonatal availability of 3′SL (Pisa et al., 2021) and 6′SL (Hauser et al., 2021) may impair attentional capabilities later in adulthood. As a biological mechanism underlying the observed affected phenotype, we propose that the reduced availability of sialic acid, caused by the lack of the most abundant sources of sialic acid in the newborn [i.e., 3′SL and 6′SL, see, respectively, Pisa et al. (2021) and Hauser et al. (2021)], impacts neuroanatomical pathways, affecting the development of neural circuits and resulting in an alteration of attention and memory. In our previous study, we observed that WT mice exposed to milk without 6′SL exhibited an altered expression of several genes associated with myelination in the pre-frontal cortex. Specifically, evaluating gene expression, we observed altered expression of genes responsible for the formation and patterning of neuronal circuits in the pre-frontal cortex region. Moreover, the gene expression was evaluated in two different time windows to assess potential temporal selectivity. With this experimental design, we were able to observe that the altered expression of genes directly involved in brain development appears only during the early stage of life. These results suggest that the lack of 6′SL, as a source of sialic acid, may induce a reduction in sialylated binding targets for the myelin-associated glycoprotein, resulting in an alteration of the formation of new neural circuits (Bode, 2012; Schnaar et al., 2014). Then, using a wide-ranging approach, through the analysis of gut microbiota and plasma metabolomics, we observed that the aforementioned behavioral deficits may be also correlated with alterations in the serotoninergic system. HMOs are known to contribute to the development of the gut microenvironment, promoting the colonization of some commensal bacteria (e.g., Bifidobacteria) and acting as prebiotics (Hennet et al., 1998). When assessing the microbiota of our experimental subjects, we found an altered concentration of specific species of microbes in the gut of mice exposed to selective deprivation of 6′SL. Specifically, we showed that lactational deprivation of 6′SL resulted in a lower abundance of Firmicutes species, one of the phyla involved in the tryptophan metabolism (Bode, 2012). The consequence of this decreased concentration in Firmicutes species is an alteration in the tryptophan metabolism of mice exposed to deprivation of 6′SL, confirmed by the plasma metabolomic analysis (Hauser et al., 2021). While these findings were generated for mice receiving milk with or without 6′SL, it has been hypothesized that the role of 3′SL and 6′SL is to provide mice with sialic acid (Wang, 2012), and thus in our dKO experiment, similar pathway could be impacted.

Although the focus of this study was to assess the effect of a specific time-limited reduced availability of 6′SL and 3′SL on the development of executive functions, our experimental design also allowed us to observe the potential effects of *St3Gal4* and *St6Gal1* deletion *per se* on cognitive functions. Albeit not directly applicable on a translational perspective (e.g., these mutations are not common in humans), it is worth discussing this result to further understand the potential mechanisms underlying the observed phenotype. The deletion *per se* of the genes responsible for the synthesis of both oligosaccharides had marginal effects whereby it only affected long-term spatial memory and did not impair other types of memory and attentional capabilities. This effect may be related to the fact that these subjects are exposed to the physiological availability of 3′SL and 6′SL, partially in contrast with the GENE + MILK group that are reared in absence of these oligosaccharides. Our results showed that, compared to CTRL mice, GENE + MILK mice were indistinguishable in attentional capabilities and recognition memory, while they were impaired in spatial memory and pre-pulse inhibition. This result is partly overlapping with our previous findings (Hauser et al., 2021; Pisa et al., 2021), wherein we observed that the depletion of the genes responsible for the secretion of 3′SL (Pisa et al., 2021) or of 6′SL (Hauser et al., 2021) in milk did not result in impaired cognitive function. To explain this unexpected phenomenon, we referred to the match/mismatch hypothesis as a potential explanation for the lack of impairments in KO mice reared to KO dams. Specifically, we started from the observation that while the absence of the St6Gal1 gene induced a reduction in host gut sialylation (offspring genotype force) (Hennet et al., 1998), 6’SL-poor milk resulted in an alteration of the gut microbiota (maternal genotype force) (Hauser et al., 2021). We then hypothesized that when these driving forces operate in opposite directions (i.e., WT mice with control gut sialylation receiving milk without sialylated oligosaccharides, or KO mice with reduced gut sialylation receiving milk with sialylated oligosaccharides) the result is a maladaptive microbiota, which in turn impacts negatively on brain functions (mismatch). Conversely, in CTRL and GENE + MILK mice both the host glycosylation and milk sialylation act in the same direction, ultimately resulting in a different, but not maladaptive, microbiota (match). While this hypothesis may explain the lack of differences between CTRL and GENE + MILK mice in our study, it does not explain the observed differences in spatial memory and pre-pulse inhibition. With respect to the latter, we suggest that the fact that both oligosaccharides (3′SL and 6′SL) are dysfunctional (instead of only one of them) may induce effects that extend beyond gut colonization. Specifically, we hypothesize that the absence of both oligosaccharides resulted in much lower concentrations of sialic acid, a component known to be essential for brain maturation (Wang et al., 2003), compared to our previous work (Hauser et al., 2021; Pisa et al., 2021) and ultimately affected the individual phenotype.

### 4.2. Exogenous supplementation of 3′SL and 6′SL affects the reproducibility of the phenotype

Our strategy to orally supplement 3′SL and 6′SL during lactation in mice cross-fostered to KO dams, which provided milk without 6′SL and with low levels of 3′SL, abolished the effects we observed in the first experiment. We therefore were not able to evaluate if an exogenous supplementation of 3′SL and 6′SL, only during the lactational period, may compensate for the observed deficits. Specifically, WT mice exposed to a milk poor of 3′SL and 6′SL, receiving a vehicle treatment, failed to show the previously observed phenotype. The subjects did not differ from control mice in memory or attentional capabilities. This finding does not allow us to discuss the second part of the results, in which mice exposed to treatment with 3′SL and 6′SL did not show significant differences compared to control mice. We propose that the supplementation procedure, which entailed some level of maternal separation, may have eliminated potential group differences by introducing an experimental bias. This may likely affect outcomes of individual tests differently. Specifically, we observed that the supplementation procedure apparently resulted in ceiling effects in NOR and T-maze and in floor effects in PPI, Barnes maze and ASST. The influence of neonatal manipulations has been already ascertained in countless studies (e.g., Hurst and West, 2010; Christakis et al., 2012; Gerlai, 2014; Raineki et al., 2014; Kafkafi et al., 2018). In our case, we propose that the manipulation necessary to perform the supplementation procedure (e.g., maternal separation, twice daily handling, administration of liquid) may have inserted a potential confound ultimately altering the behavioral performances observed in our study.

## 5. Conclusion

We have demonstrated that the absence of sialylated oligosaccharides during the early stage of life induces impairments in cognitive functions such as memory and attention. We failed to demonstrate that exogenous supplementation of these oligosaccharides may revert the observed phenotype, but we propose that this lack of effect is due to a ceiling effect dependent on the experimental design and not to an absence of effect of the supplementation. We believe that further studies are needed to overcome the limitation that we faced in the current work. As a solution, we propose to change the supplementation method, reducing to a bare minimum the manipulation of the neonatal subjects. For example, limiting the maternal separation and the session of supplementation. Together with our previous evidence, present results highlight the pivotal role of breast-milk oligosaccharides constituents, specifically 3′SL and 6′SL, in the development of cognitive function, including executive functions (e.g., attention, working memory), albeit future studies are needed to prove that the exogenous administration of these sialylated oligosaccharides is necessary to overcome their absence during lactation.

## Data availability statement

The raw data supporting the conclusions of this article will be made available by the authors, without undue reservation.

## Ethics statement

This animal study was reviewed and approved by Institutional Animal Survey Board on behalf of the Italian Ministry of Health (license no. 91/2016-PR to SM).

## Author contributions

SM, EP, and JH designed the research and revised and consolidated the manuscript. EP and AO performed the behavioral experiments. AT and VC performed the genotyping procedure. EP wrote the first draft of the manuscript. All authors contributed to the article and approved the submitted version.
