# Supplementary material for: Long-term consequences of reduced availability and compensatory supplementation of sialylated HMOs on cognitive capabilities
Source: Front Cell Neurosci. 2023 Jan 30;17:1091890. doi: 10.3389/fncel.2023.1091890 (PMC9922896; doi:10.3389/fncel.2023.1091890)
Supplement: Supplementary file 1 [file Data_Sheet_1.pdf]

Supplementary Material

Attentional set-shifting task

To assess whether experimental subjects differed in cognitive flexibility and attentional capabilities, we performed the attentional set-shifting task. In the experiment 1, during the SD phase, MILK subjects committed more errors of CTRL group (offspring genotype: $F_{1,32}$=0.017 p-value=0.89; maternal genotype: $F_{1,32}$=0.66 p-value=0.44; interaction between offspring and maternal genotype: $F_{1,32}$=11.91 p-value=0.001, p<0.05 in post-hoc tests). In CD phase, MILK subjects committed more errors during the stage (offspring genotype: F1,32=0.17 p-value=0.78; maternal genotype: $F_{1,32}$=1.08 p-value=0.31; interaction between offspring and maternal genotype: $F_{1,32}$=5.86 p-value=0.02, p<0.05 in post-hoc tests). During CDR, IDs and EDs phases, there was no difference between groups in errors (data not shown, see supplementary figure 4a). Albeit these results, in the experiment 2 we did not observe any differences between groups during all the phases of the test in errors committed (see supplementary figure 4b).

Novel object recognition task

**Group**	**Time with novel object**	**Time with familiar object**
CTRL	88 ± 12.26	36 ± 5.9
MILK	86.5 ± 20.63	49.7 ± 9.5
GENE	84.38 ± 15.77	47.87 ± 8.7
GENE + MILK	106 ± 11.66	41.21 ± 8.03

Supplementary Table 1. Time spent (seconds) with novel and familiar object during the test phase of the novel object recognition task for experiment 1(data are expressed with mean ± SEM).

**Group**	**Time with novel object**	**Time with familiar object**
CTRL - H_2O	167.3 ± 14.24	185.19 ± 16.09

MILK - H_2O	160.26 ± 12.29	166.41 ± 12.79
CTRL - SL	171.26 ± 12.29	176.65 ± 18.75
MILK - SL	169.28 ± 16.19	156.26 ± 11.29

Supplementary Table 2. Time spent (seconds) with novel and familiar object during the test phase of the novel object recognition task for experiment 2 (data are expressed with mean ± SEM).

Supplementary Figure 1. Maternal care was assessed, during the first 10 days of life, through focal sampling over two one-hour periods. In each one-hour period, dams were observed 20 times at regular 3-min intervals. Data are expressed as average hourly frequency of nursing. Inset: main effect of the genotype.

Supplementary Figure 2. Main effect of the maternal genotype of experiment 1 in percentage of alternations during the T-maze. - $p < 0.05$ significantly different from WT genotype in post-hoc tests.

Supplementary Figure 3. Main effect of the offspring genotype of experiment 1 in time spent in target zone during one week probe trial of the Barnes maze. - $p < 0.05$ significantly different from WT genotype in post-hoc tests.

Supplementary Figure 4. Number of errors committed in all phases in experiments 1 (a) and 2 (b). * $p < 0.05$ significantly different from CTRL group.

Supplementary Figure 5. Main effect of the offspring genotype of experiment 1 in the blood glucose concentration. - $p < 0.05$ significantly different from WT genotype in post-hoc tests.

Supplementary Figure 6. Main effect of the maternal genotype of experiment 2 in the blood glucose concentration. - $p < 0.05$ significantly different from WT genotype in post-hoc tests.
