# Supplementary figures and images for: Long-term consequences of reduced availability and compensatory supplementation of sialylated HMOs on cognitive capabilities
Source: Front Cell Neurosci. 2023 Jan 30;17:1091890. doi: 10.3389/fncel.2023.1091890 (PMC9922896; doi:10.3389/fncel.2023.1091890)

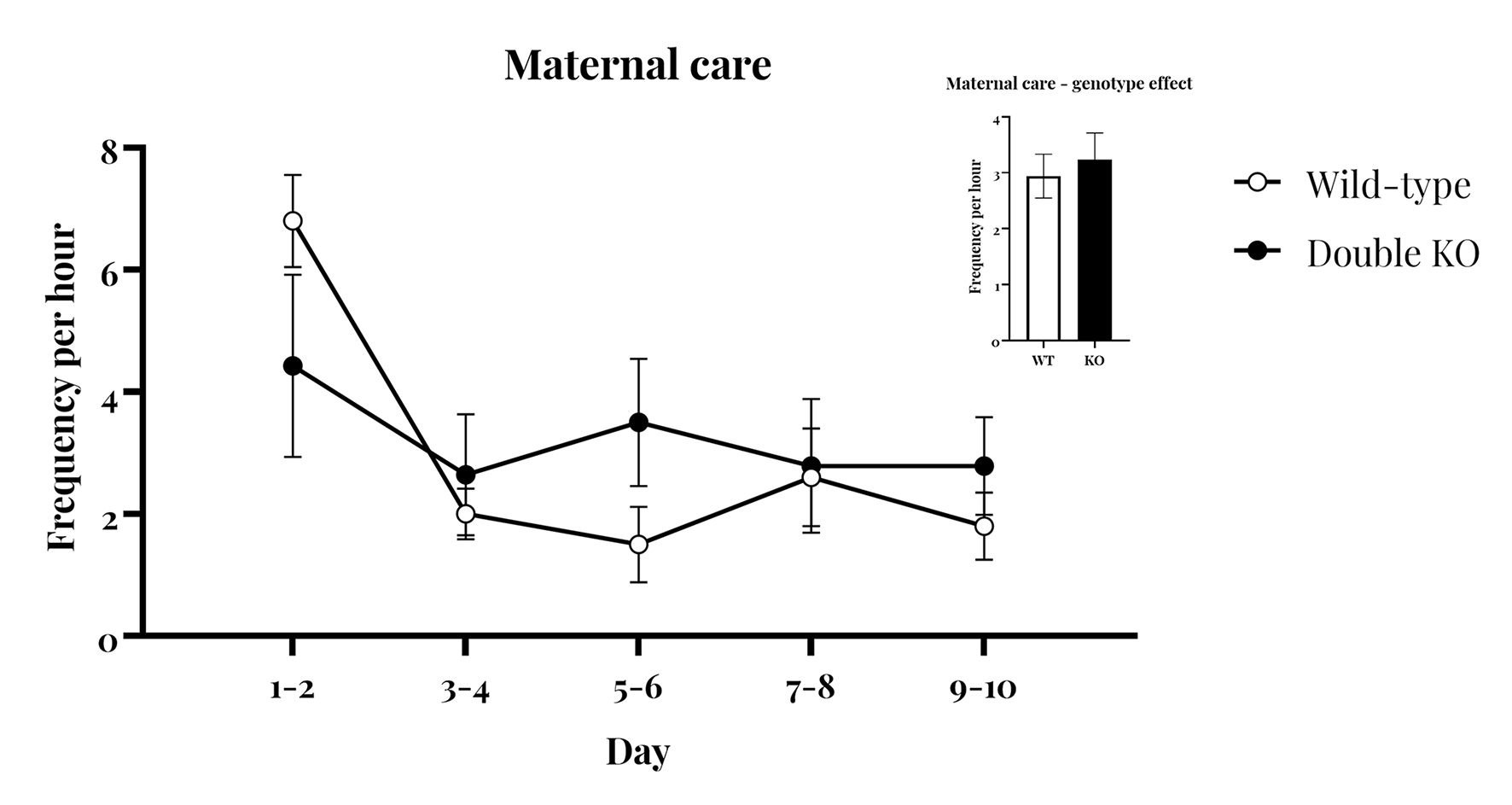

Supplement: Supplementary file 2 [file Image_1.TIF]

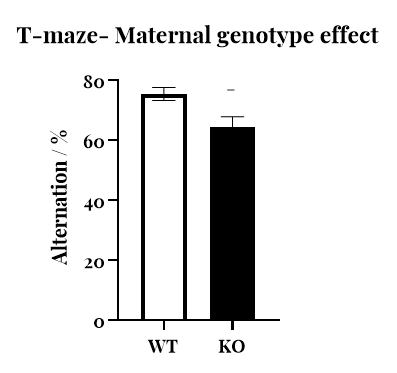

Supplement: Supplementary file 3 [file Image_2.TIF]

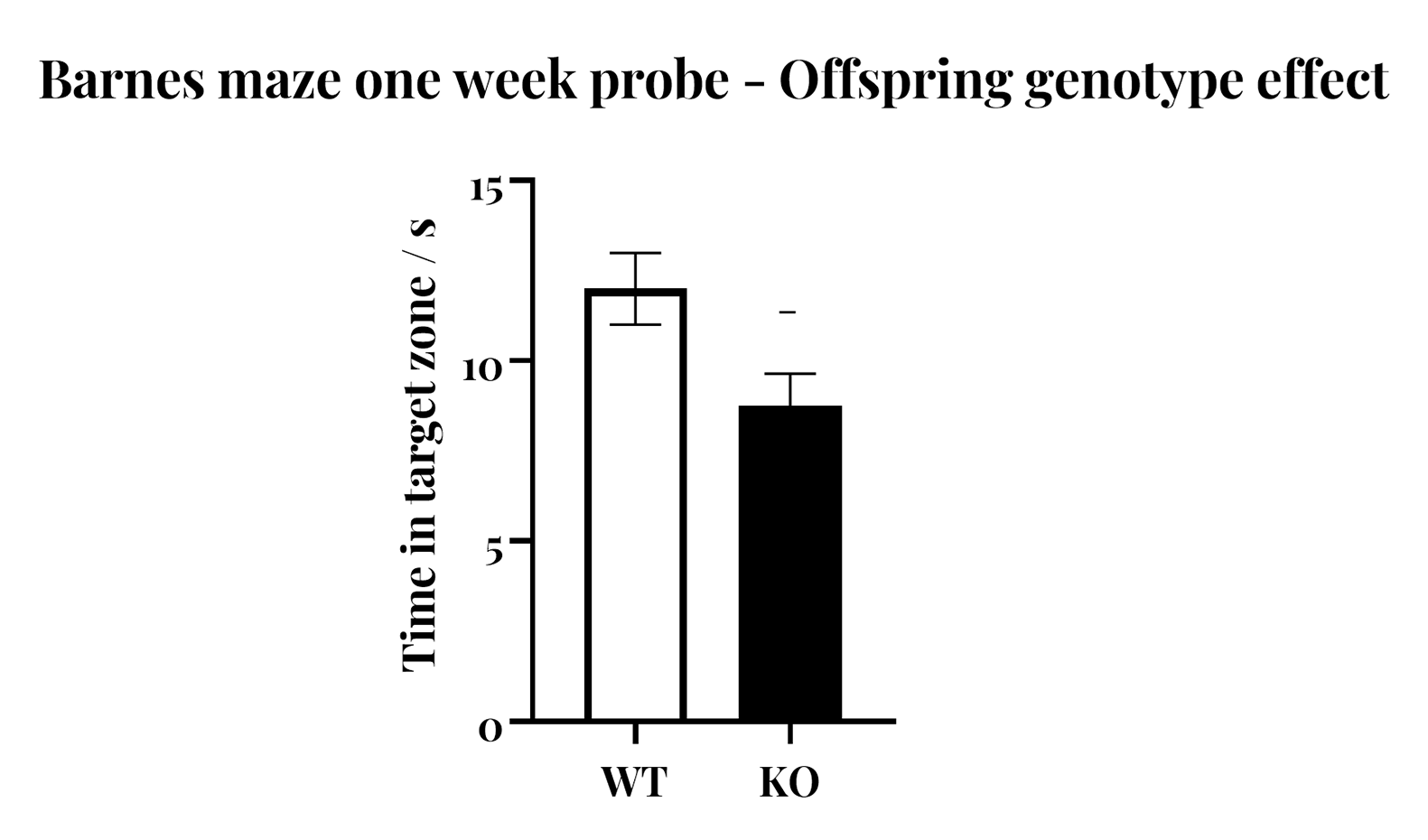

Supplement: Supplementary file 4 [file Image_3.TIF]

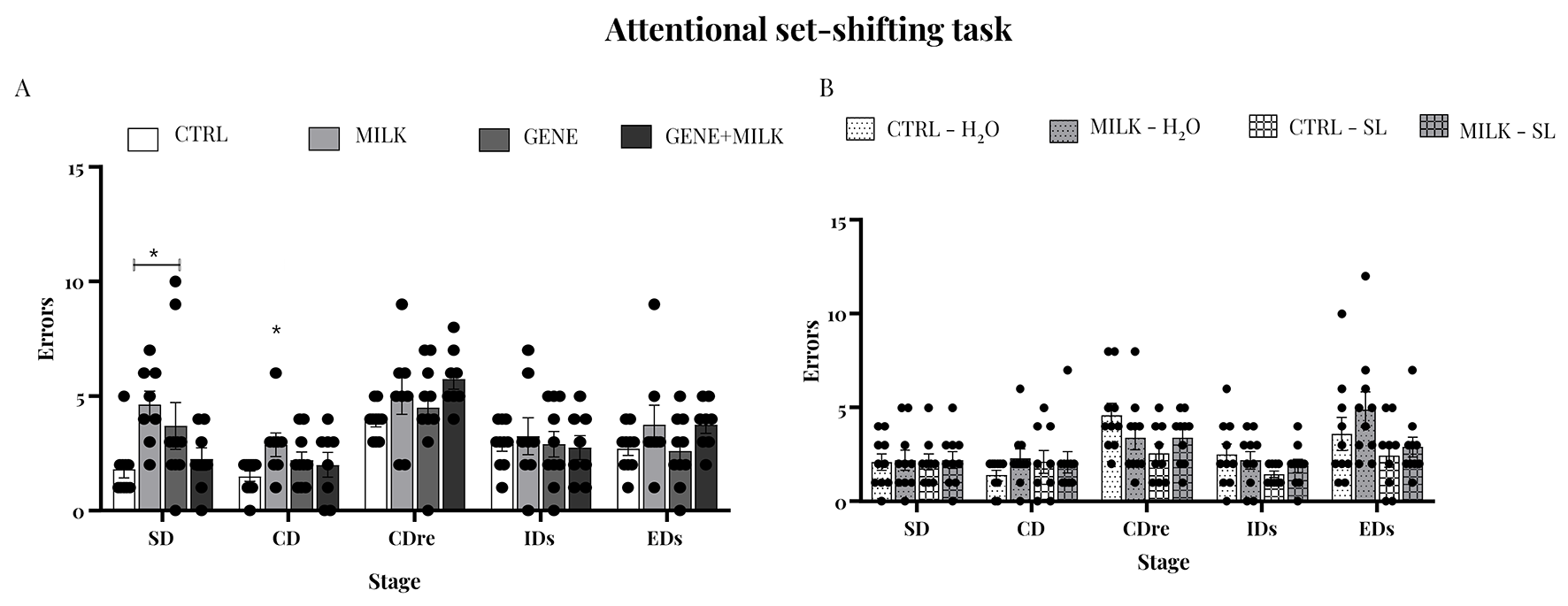

Supplement: Supplementary file 5 [file Image_4.TIF]

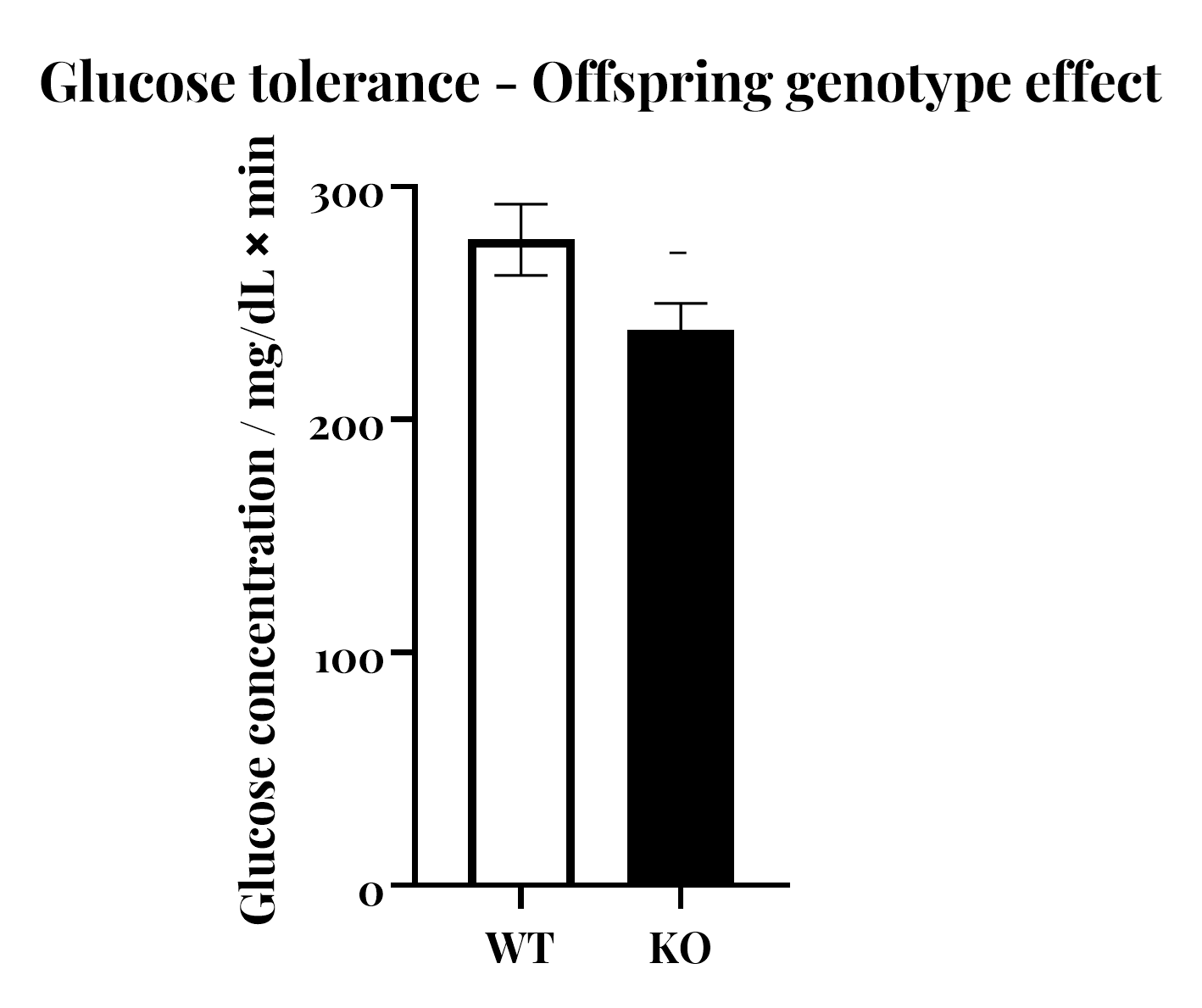

Supplement: Supplementary file 6 [file Image_5.TIF]

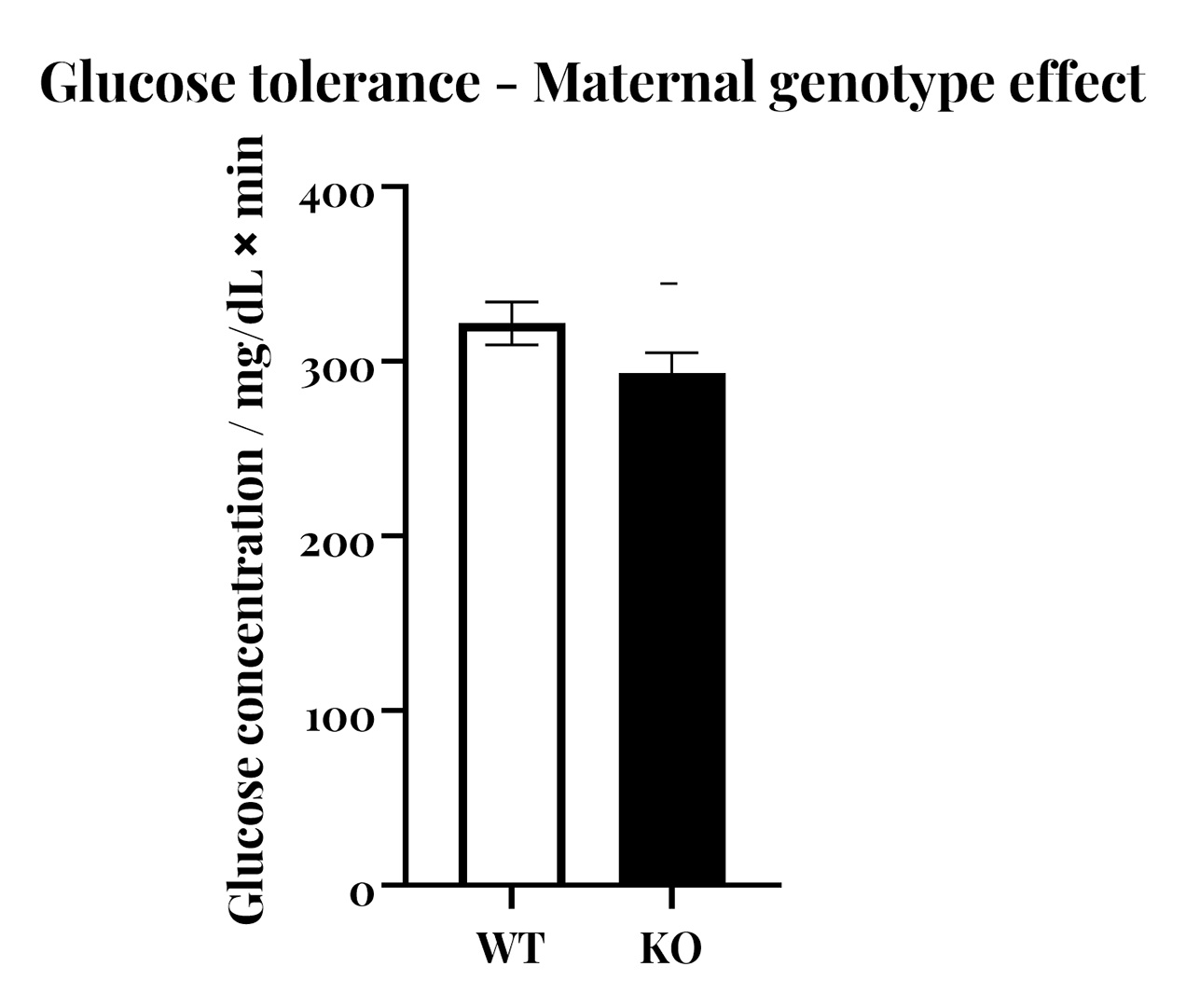

Supplement: Supplementary file 7 [file Image_6.TIF]
